# Serum-binding properties of isosteric ruthenium and osmium anticancer agents elucidated by SEC–ICP–MS

**DOI:** 10.1007/s00706-018-2280-1

**Published:** 2018-08-25

**Authors:** Matthias H. M. Klose, Anna Schöberl, Petra Heffeter, Walter Berger, Christian G. Hartinger, Gunda Koellensperger, Samuel M. Meier-Menches, Bernhard K. Keppler

**Affiliations:** 1 Institute of Inorganic Chemistry, University of Vienna, Waehringer Strasse 42, 1090 Vienna, Austria; 2 Research Cluster ‘Translational Cancer Therapy Research’, University and Medical University of Vienna, Vienna, Austria; 3 Department of Analytical Chemistry, University of Vienna, Waehringer Strasse 38, 1090 Vienna, Austria; 4 Department of Medicine I and Comprehensive Cancer Centre of the Medical University, Institute of Cancer Research, Medical University of Vienna, Vienna, Austria; 5 School of Chemistry, University of Auckland, Private Bag 92019, 1142 Auckland, New Zealand

**Keywords:** Antitumor agents, Arene complexes, Bioinorganic chemistry, Inductively coupled plasma, Mass spectrometry

## Abstract

**Abstract:**

Size-exclusion chromatography–inductively coupled plasma–mass spectrometry (SEC–ICP–MS) was used to study the serum-binding preferences of two metallodrugs with anticancer activities in vivo, namely the organoruthenium compound plecstatin-1 and its isosteric osmium analog. The complexes were administered intraperitoneally into mice bearing a CT-26 tumor. Comparing the total metal content of mouse whole blood and serum underlined that the metallodrugs are mainly located in serum and not in the cellular fraction of the blood samples. In mouse serum, both compounds were not only found to bind extensively to the serum albumin/transferrin fraction but also to immunoglobulins. Free drug was not observed in any of the samples indicating rapid protein binding of the metallodrugs. These findings were validated by spiking human serum with the respective compounds ex vivo. An NCI-60 screen is reported for the osmium analog, which revealed a relative selectivity for cancer cell lines of the ovary and the central nervous system with respect to plecstatin-1. Finally, a COMPARE 170 analysis revealed disruption of DNA synthesis as a possible treatment effect of the osmium-based drug candidate.

**Graphical abstract:**

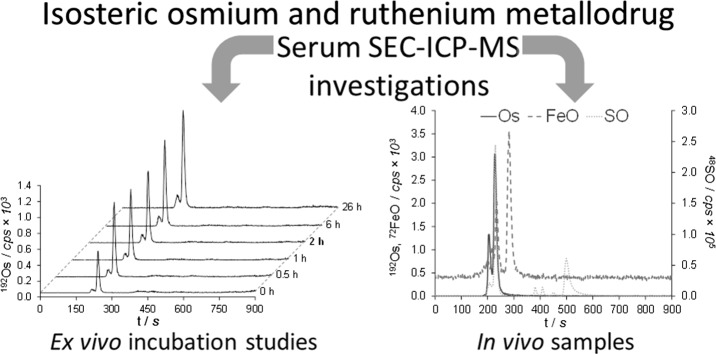

## Introduction

Coupling liquid chromatography (LC) to inductively coupled plasma–mass spectrometry (ICP–MS) is a niche technique, despite its obvious advantages [1, 2]. LC–ICP–MS offers chromatography-based separation efficiency combined with the low detection limits, wide dynamic range, and multi-element detection capability of ICP–MS as a powerful analytical method for tracing metals and other elements in the sub-ppb range and in complex matrices. The hyphenation is facilitated by similar flow rates of the LC and ICP–MS instruments [2].

In the field of inorganic anticancer drug research, LC–ICP–MS can be used to monitor protein interactions and to investigate compound stability [1, 3]. Studies on the metabolism of gold-based auranofin in human blood were among the first applications of LC–ICP–MS in the research field [4]. Platinum anticancer agents are arguably the most widely investigated metallodrugs and their DNA targeting is well established [3]. Since they are administered intravenously, potential first binding partners in vivo are blood constituents, involving soft donor atom ligands such as thiol-containing proteins to which the activated platinum anticancer agents may bind, e.g., human serum albumin (HSA). As HSA and other macromolecules can accumulate in tumor tissue, serum protein–drug conjugates are exploited as drug-delivery systems mostly by means of surface-exposed cysteines [5, 6]. This is exemplified by abraxane, a clinically applied albumin–paclitaxel conjugate for the treatment of breast cancer [7], as well as investigational platinum [8–10] and ruthenium conjugates [11]. Early investigations involving LC–ICP–MS focused not only on the interaction of cisplatin, carboplatin, and oxaliplatin with HSA, human serum transferrin (hTf), immunoglobulin G (IgG), metallothionein (MT), glutathione (GSH) but also plasma, serum, and whole blood [3, 12–20]. In addition, different chromatographic separation techniques were employed in combination with ICP–MS, including hydrophilic interaction liquid chromatography (HILIC) [19], reversed-phase (RP) chromatography [13, 18, 21, 22], and size-exclusion chromatography (SEC) [12, 14–17, 23, 24]. Recently, LC–ICP–MS studies were successfully applied to study the intracellular distribution of intact cisplatin [25] or platinum(IV) drug candidates in fractionated whole blood [26].

Next-generation anticancer metallodrugs based on ruthenium and osmium show great promise because they follow novel modes of action compared to classical platinum drugs, which are still often not elucidated in detail [27]. Thus, LC–ICP–MS studies were also carried out for this compound class, including the clinically investigated ruthenium coordination compounds KP1019 and IT-139 (formerly known as NKP-1339) [24]. The plasma-binding characteristics of KP1019 in vivo were investigated revealing HSA as the preferred binding partner. A subsequent report confirmed this finding also for the more soluble IT-139 [28]. Moreover, SEC–ICP–MS measurements were carried out to study the interaction of RAPTA-T, an organometallic ruthenium(arene) compound bearing a phosphaadamantane ligand, with HSA and hTf at different concentrations [5]. While RAPTA-T preferred binding to hTf over HSA, cisplatin was found to interact with both proteins to a similar extent. Similar investigations were also performed on gadolinium [29, 30], titanium [31], and tungsten compounds [32], among others [3].

**Fig. 1:**
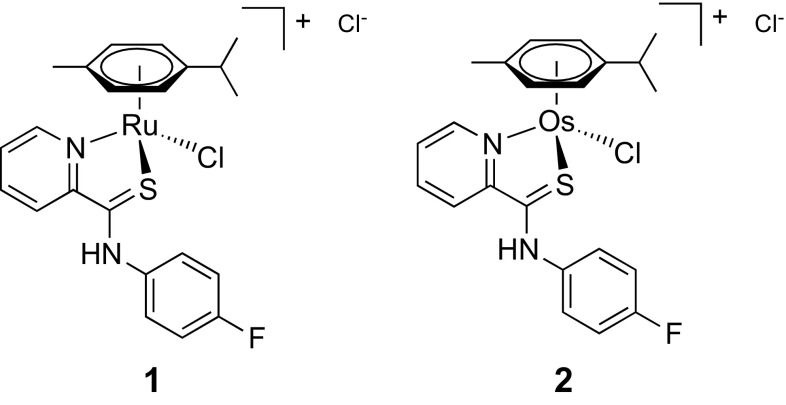
Chemical structures of the investigated organometallic drug candidates containing ruthenium (**1**, plecstatin-1) and osmium (**2**)

We recently discovered a compound family of metal(arenes) based on *S*,*N*-chelating pyridinecarbothioamides as potential anticancer agents, where the metal is either ruthenium or osmium [33]. Importantly, we identified plectin, a scaffold protein and cytolinker, as the main target of the lead compound [chlorido(η^6^-*p*-cymene)(*N*-(4-fluorophenyl)-2-pyridinecarbothioamide)ruthenium(II)] chloride, termed plecstatin-1 (**1**, Fig. 1) [34]. This compound showed distinct tumor-inhibiting effects against the CT-26 colon cancer and the B16 melanoma tumor models after oral administration [34, 35]. An additional survival experiment in the CT-26 tumor model with **1** and its osmium analog **2** confirmed tumor-inhibiting effects after treatment termination (day 14), however, mice treated with **2** showed a sustained partial tumor inhibition up to day 21 [36].

To characterize similarities and differences of this pair of promising isosteric compounds, we successfully developed methods for the element-specific quantification of osmium by ICP–MS, which refused previous quantification attempts due to its tendency to form volatile OsO_4_ during sample preparation [35]. LA–ICP–MS studies were also performed on these compounds to assess their spatial distributions in tumors and organ tissues of treated tumor-bearing mice [36]. To further elucidate the influence of the metal center of **1** or **2** on their distinct biological effects, we report here on the SEC–ICP–MS analysis of serum samples collected from tumor-bearing mice, which were treated i.p. with a single dose of either **1** or **2**. We compared the total metal concentration in serum to whole blood and characterized likely binding partners in serum. The results from the mouse serum samples were validated by spiking human serum ex vivo with the respective compounds. Finally, **2** was evaluated in the NCI-60 cell line panel and compared to **1**. To the best of our knowledge, this is the first study on serum-binding properties of an osmium-based anticancer metallodrug by SEC–ICP–MS.

## Results and discussion

Recently, we reported on the successful quantification of osmium-based metallodrugs by ICP–MS by means of a stabilizing solution capable of inhibiting OsO_4_ formation after microwave digestion [35]. This allowed us to investigate more closely the distribution of a pair of isosteric drug candidates based on ruthenium (**1**) and osmium (**2**) in organs of treated mice by ICP–MS and their spatial distribution in organs and tumors by LA–ICP–MS [35, 36]. Although the two compounds showed distinct antitumor activity in vivo, these studies did not reveal considerable differences in the biodistribution of **1** and **2**. For this purpose, it is of interest to determine the fraction of both metallodrugs in mouse serum with respect to whole blood and their binding preferences towards serum constituents, e.g., serum albumin, transferrin (66–80 kDa) or immunoglobulins (> 100 kDa). The latter can be achieved by SEC–ICP–MS [37].

Of the six serum samples from mice treated with **1** or **2**, five (Ru: *n* = 3; Os: *n* = 2) were diluted with either 3% HNO_3_ (Ru) or with stabilization solution in 3% HNO_3_ (Os) with a factor of 1:250 and the total metal contents were determined. Due to the availability of a limited volume of the third Os-serum sample, it was used solely for SEC–ICP–MS analysis. The determined metal contents were in the low µg g^−1^ range in agreement with previous studies [36]. Intriguingly, an approximate twofold higher metal content in serum compared to whole blood was observed for both compounds indicating that they bind nearly exclusively to constituents in serum and not to blood cells, when considering that whole blood consists of roughly 50–60% serum (Table 1). Our findings paralleled previous results with IT-139 [38]. In contrast, oxaliplatin was found mainly bound to erythrocytes [39], while some platinum(IV) drug candidates displayed tunable accumulation in blood cells [26]. It may be hypothesized that charged metallodrugs prefer binding to serum constituents, while neutral metallodrugs may tend to accumulate in the blood cell fraction of whole blood.

**Table 1:** Determination of total metal content in µg g^−1^ in serum and whole blood by ICP–MS

	**1** (Ru)	**2** (Os)	Reference
Serum	2.61 ± 0.85	4.63	
Blood	1.08 ± 0.20	1.71 ± 0.25	[36]

Hyphenating SEC to the ICP–MS operated in oxygen mode enables monitoring not only the respective metal center of a metallodrug but also the sulfur trace via ^48^SO^+^, which is usually mass shifted to avoid the isobaric ^16^O^16^O^+^ interference on the ^32^S^+^ isotope. This is especially useful for metallodrug–protein interaction studies since proteins may comprise the sulfur-containing amino acids l-methionine and l-cysteine. However, interferences may occur also for metal isotopes, especially, if they are characterized by a low mass number [3]. Ruthenium could potentially suffer from argon, nickel, chlorine, and rubidium interferences, and therefore, at least two isotopes were recorded to control for false-positive signals. This is especially important in biological matrices [3]. In contrast to ruthenium, osmium has no interferences originating from a biological background. However, since osmium is prone to oxidation, the respective mono-, di-, tri-, and tetra-oxygen species of ^192^Os^+^ were recorded in addition to the ^189^Os^+^ and ^192^Os^+^ ions.

**Fig. 2:**
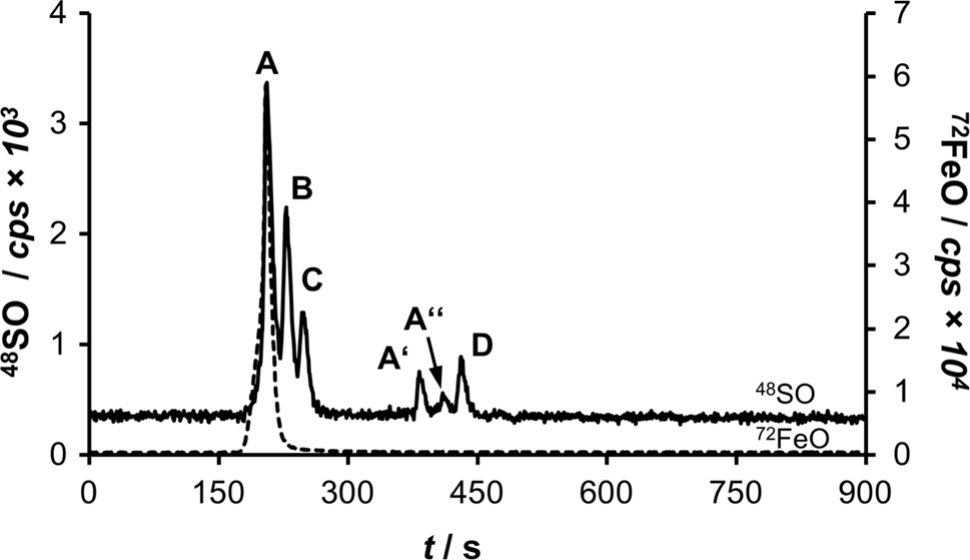
SEC–ICP–MS analysis of the size standard mixture showing the ^48^SO^+^ and ^72^FeO^+^ (dashed line) traces. The peaks are as follows: ferritin (A and fragments thereof A′, A″), albumin (B), ovalbumin (C), and methionine (D)

A mixture of ferritin (440 kDa), bovine albumin (66 kDa, BSA), ovalbumin (43 kDa), and methionine (102 Da) was used as the size standards to calibrate SEC–ICP–MS runs. Ferritin was chosen as a dead time marker, which exceeds the separation capacity of the employed SEC column (< 80 kDa). Recording the ^72^FeO^+^ trace in addition to the ^48^SO^+^ trace unambiguously identified the ferritin peak in this setting (Fig. 2). The total run time was 900 s and the serum constituents eluted between 200 and 500 s.

**Fig. 3:**
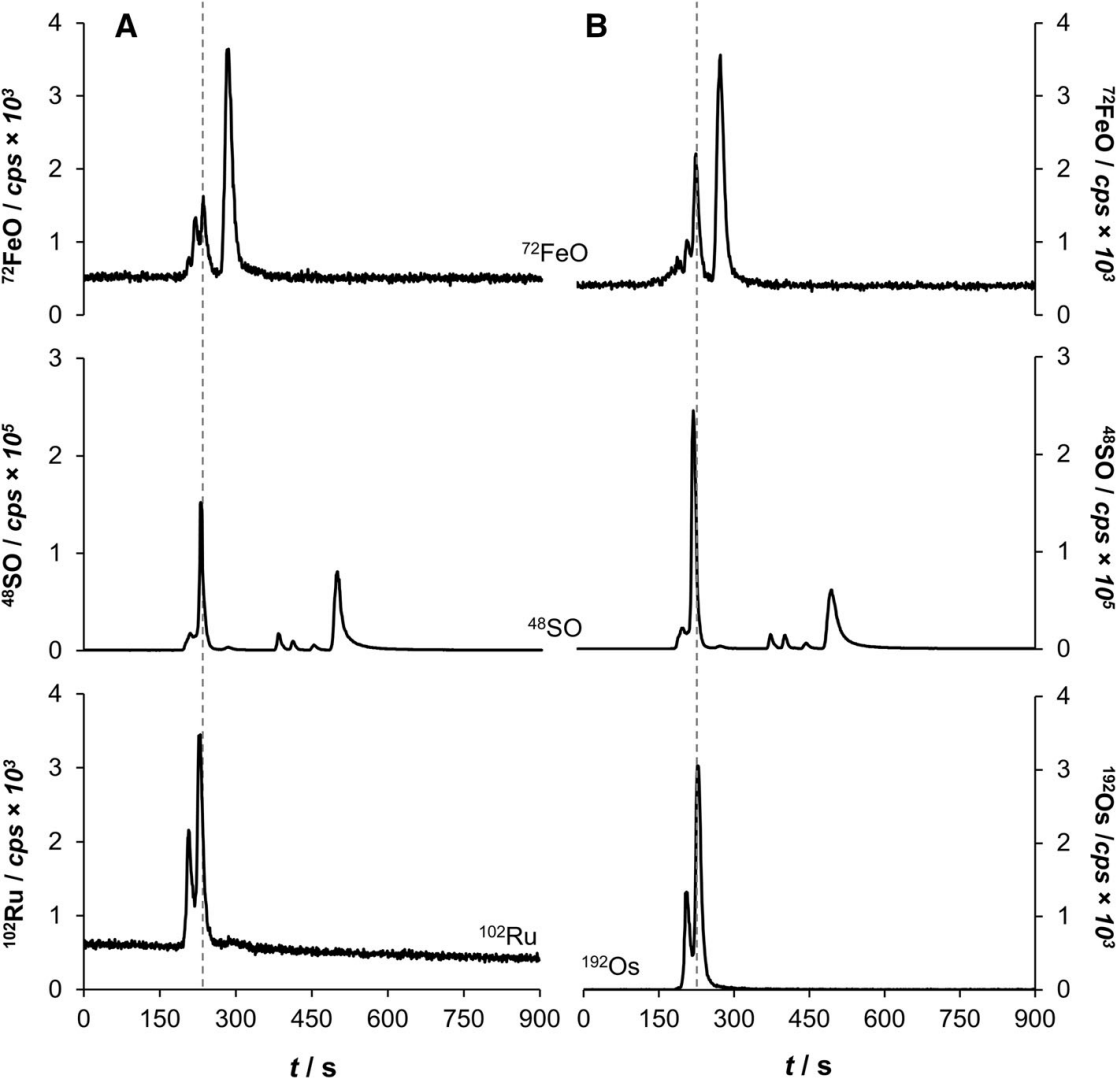
Representative chromatograms of serum samples of mice treated with either **1** (**a**) or **2** (**b**) and measured by SEC–ICP–MS. Illustrated are the ^72^FeO^+^, ^48^SO^+^ and ^192^Os^+^, or ^102^Ru^+^ traces

Six serum samples of treated mice with **1** or **2** (Ru, Os: each *n* = 3) were diluted 1:20 in mobile phase (see experimental section) and 2 mm^3^ were injected onto the SEC column. All samples treated with **1** showed the same binding interactions with two main peaks in the ^102^Ru^+^ trace (Fig. 3). The major peak with a retention time of 228 s corresponded to the fraction containing both albumin and transferrin, while the minor peak at 204 s close to the dead time indicated immunoglobulin-bound ruthenium. The area ratio of the two peaks was 3:1 in favor of the albumin/transferrin fraction, with a deviation of ± 2% among biological replicates. Free compound **1** was not observed in any of the mouse serum samples. Since the treatment time was 2 h, this indicated that serum protein binding in vivo occurred quickly. The osmium-based **2** showed a very similar binding pattern in serum according to the ^192^Os^+^ trace. Again, the main peaks were identified as albumin/transferrin and immunoglobulin adducts in a ratio of 3:1 in favor of the albumin/transferrin fraction, with a deviation of 1% among biological replicates. No free **2** was observed in the low mass area of the chromatogram (Fig. 3).

The ^72^FeO^+^ trace revealed a significant iron peak at roughly 300 s indicating free hemoglobin from disrupted erythrocytes during sample collection. Moreover, the ^48^SO^+^ trace revealed a considerable peak at approximately 500 s, eluting after the l-methionine standard (112 Da). Dissolving compound **2** with and without dimethylsulfoxide (DMSO) gave evidence that this peak is most likely the result of DMSO addition (data not shown). Finally, the same chromatographic profiles were obtained for **2** when analyzing the shifted masses on ^208^OsO^+^ and ^224^OsO_2_^+^ with about half to a third of the counts compared to the parent ^192^Os^+^ trace, while the ^240^OsO_3_^+^ and ^256^OsO_4_^+^ traces did not reveal any signal.

**Fig. 4:**
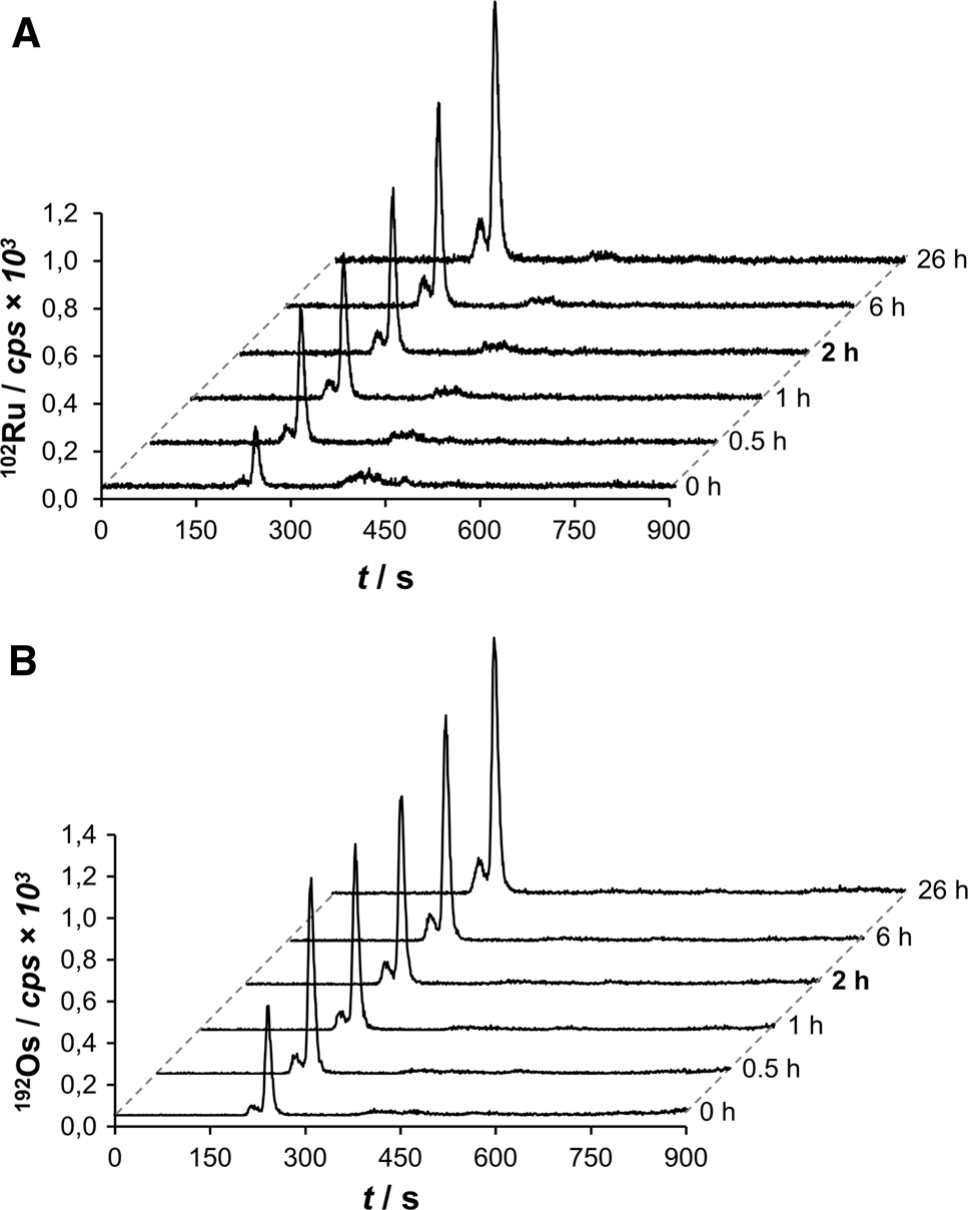
Incubation studies of human serum spiked with **1** (**a**) and **2** (**b**) by SEC–ICP–MS. The 2 h run is highlighted due to the similarity to the in vivo experiment

To validate these findings, the anticancer compounds **1** and **2** were spiked to human serum and their respective binding profiles were evaluated by SEC–ICP–MS in a time-dependent manner ex vivo. These mixtures were incubated at 37 °C and were identically processed to a final concentration of approximately 0.5 µM. Aliquots were analyzed after 0 min, 30 min, 1, 2, 6, and 26 h, respectively. Again, rapid binding of both **1** and **2** to the albumin/transferrin fraction and immunoglobulins was observed, which mirrors perfectly the findings from the mouse experiments. The intensity of the metal-bound albumin/transferrin fraction seemed to increase over time (Fig. 4), while the ratio of the peak areas for Ru at albumin/transferrin:immunoglobulin remained fairly constant ranging from 6.7:1 (0 min) to 4.5:1 (26 h) for **1** in favor of the albumin peak. Compound **2** displayed very similar ratios, which changed from 8.1:1 (0 min) to 6.7:1 (26 h). Interestingly, binding of both compounds levelled off after 1 h of incubation. Thus, the similarity in binding behavior and binding kinetics of the isosteric ruthenium and osmium compounds suggested that binding is rather driven by electrostatic and non-covalent interactions than by hydrolysis and subsequent coordination of the respective metal center to the proteins.

**Fig. 5:**
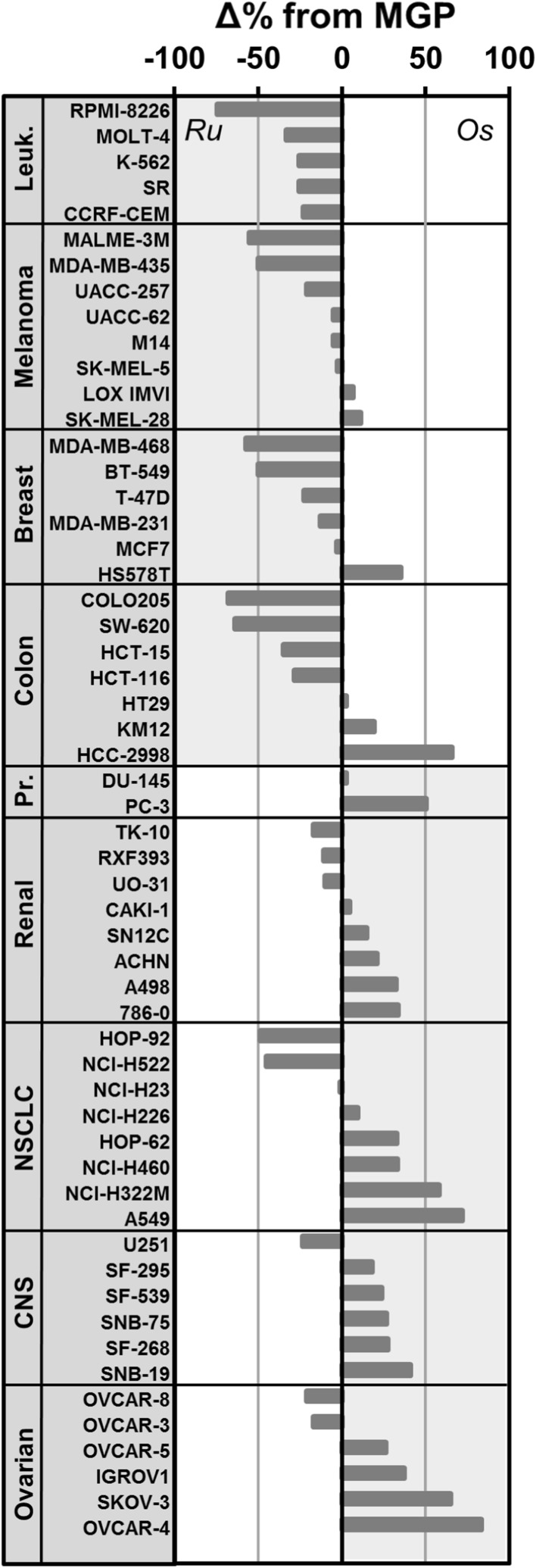
Difference of growth inhibition in % of mean growth percentage (MGP) between **1** (Ru) and **2** (Os) in the NCI-60 cancer cell line panel. The cancer cell lines are grouped according to tumor type from ruthenium-selective activity (top) to osmium-selective activity (bottom). *Leuk*. leukemia, *NSCLC* non-small cell lung cancer, *CNS* central nervous system, *Pr.* prostate

Osmium-based **2** (NSC No. 776416) was assessed with respect to its cytotoxicity at 10 µM in the NCI-60 cancer cell line panel [40]. The one-dose study provides an estimate of the inhibitory effect of the compound on cancer cell growth. The results for **2** can be directly compared to that of **1** [34]. Compound **2** was overall less potent than **1** (NSC No. 776415) as underlined by a mean growth percentage (MGP) of 70 and 48%, respectively. However, the relative pattern of growth inhibition was similar for both compounds. The compounds featured increased activity in leukemia and breast cancer cell lines with respect to their MGP. Interestingly, when comparing the relative deviation from the MGP as a percentage to directly compare the growth inhibition profiles of **1** and **2**, it emerged that **2** showed selective activity across cancer cell lines of the ovary and the central nervous system, while **1** was more selective across leukemia and melanoma cell lines (Fig. 5).

Finally, a COMPARE 170 analysis revealed caracemide (S253272, 0.63) and fluorodopan (S73754, 0.62) as the top two significant correlators for **2**. Caracemide inhibits ribonucleotide reductase resulting in decreased DNA synthesis [41, 42], while fluorodopan is an alkylating agent [43]. This indicates that DNA synthesis might be disrupted upon treating cancer cells with **2**. The plectin-targeting **1** did not feature significant correlators in the COMPARE 170 analysis. Investigations into the mode of action of **2** are thus warranted since it was previously shown that the compound would bind preferentially to histone proteins in the nucleosome core particle and not to DNA [33]. Consequently, DNA synthesis would likely be inhibited independently of direct metallodrug–DNA interactions.

## Conclusion

This study reports on the serum-binding properties of two isosteric ruthenium and osmium drug candidates, namely plecstatin-1 and its osmium analog. The compounds were found to interact mainly with serum constituents in blood of tumor-bearing mice treated with the respective metallodrug. Mouse serum was then analyzed by SEC–ICP–MS. Both drug candidates interacted quickly with the albumin/transferrin fraction and immunoglobulins in mouse serum in a ratio of 3:1 in favor of the albumin/transferrin fraction. This finding was validated by incubating the compounds ex vivo with human serum. An NCI-60 screen of the osmium-based drug candidate revealed a selective relative growth inhibition across cancer cell lines of the ovary and the central nervous system when directly compared to plecstatin-1. Finally, COMPARE 170 analysis indicated that the osmium-based drug candidate may cause inhibition of DNA synthesis as a treatment effect, which contrasts the plectin-targeting ruthenium analog. Finally, the similar biodistribution and serum protein-binding preferences of the isosteric metal-based anticancer agents indicate that pharmacokinetics are probably dictated by the ligand sphere, while the modes of action may be directly influenced by the metal center.

## Experimental

### Safety considerations

Oxidative work-up of osmium samples may release volatile and toxic OsO_4_. It is recommended to use appropriate venting systems and work strictly in fume hoods.

### Chemicals

The compounds [chlorido(η^6^-*p*-cymene)(*N*-fluorophenyl-2-pyridinecarbothioamide)M(II)] chloride, with M=Ru (**1**) or Os (**2**) were synthesized according to a previously published method [33]. Ultrapure water (18.2 MΩ cm, Milli-Q Advantage, Darmstadt, Germany) was used for all dilutions for ICP–MS measurements. Nitric acid (≥ 69%, TraceSELECTs, Fluka, Buchs, Switzerland) was used without further purification. Osmium, ruthenium, rhenium, and indium standards for ICP–MS measurements were purchased from CPI International (Amsterdam, The Netherlands). Acetic acid (Rotipuran Supra 100%) was purchased from Lactan, ammonia (25% supra pure) from VWR, bovine serum albumin (BSA), human serum, methionine and ferritin from Sigma-Aldrich, ovalbumin from GE Healthcare and DMSO was obtained from Acros organics. All other reagents and solvents were obtained from commercial sources and were used without further purification.

### Stabilization solution

The stabilization solution for Os contained equimolar amounts of ascorbic acid, thiourea and EDTA at 500 mmol dm^−3^. The solution was also used for the preparation of standards with a final nitric acid concentration of < 4% (w/w).

### Animal experiments

Experiments were carried out according to the Austrian and FELASA guidelines (BMWF-66.009/0084-II/3b/2013) for animal care and protection. Six- to eight-week-old female Balb/c mice were kept in a pathogen-free environment and every procedure was performed in a laminar airflow cabinet. CT-26 cells (5 × 10^5^ cells) were injected subcutaneously into the right flank. Three animals per group were each administered a single dose of 15 mg kg^−1^ i.p. when tumor nodules were palpable. Before administration, both compounds were individually dissolved in 10% DMSO (1.5 mg cm^−3^) and mice were administered with **1** or **2**. The animals were anesthetized after 2 h and their serum samples were collected. Blood was allowed to clot and serum was separated from cellular blood fraction by centrifugation (2×, 4000 rpm, 4 °C). Each sample was immediately ice cooled and stored at − 20 °C until measurement.

### Sample preparation

Serum samples of mice treated with the respective anticancer drug (each group contained three animals) were split in two parts. To determine the total metal content in serum, the serum samples (*n* = 5) were diluted in either 3% HNO_3_ (Ru, *n* = 3) or in stabilization solution with 3% HNO_3_ (Os, *n* = 2). We had two samples in the osmium group because of low sample volume. The dilution factor was 1:250. For SEC–ICP–MS studies, serum samples (*n* = 6) from mice were diluted 1:20 in the mobile phase (50 mM ammonium acetate) and 2 mm^3^ were injected onto the SEC column.

### ICP–MS parameters

The ICP–MS measurements were carried out on an Agilent 7500ce quadrupole ICP–MS (Agilent Technologies, Waldbronn, Germany), which was equipped with a CETAC ASX-520 autosampler (Nebraska, USA) and a MicroMist nebulizer at a sample uptake rate of approximately 0.25 cm^3^ min^−1^. The Agilent MassHunter software package (Workstation Software, version B.01.01, Build 123.11, Patch 4, 2012) was used for data processing. Nickel was used as cone material. The metals were monitored according to their respective metal center (Fig. 1). The experimental parameters were as follows: 1560 W RF power, 0.92–0.97 dm^3^ min^−1^ carrier gas, 0.22–0.27 dm^3^ min^−1^ make up gas, 15 dm^3^ min^−1^ plasma gas, 0.3 s dwell time, and 10 replicates. Doubly charged species and oxide rates were < 5%. HNO_3_ (1–3% w/w) was used as the rinsing solution.

### SEC–ICP–MS measurements

The SEC–ICP–MS measurements were carried out on an Agilent 1260 Infinity Bio-inert HPLC System (Agilent Technologies, Waldbronn, Germany) hyphenated to an Agilent 8800 ICP–MS/MS instrument (Agilent Technologies, Tokyo, Japan). The instrumental parameters are given in Table 2. SEC–ICP–MS data was recorded using the Agilent MassHunter software package (Workstation Software, Version B.01.03, 2013). Chromatographic separations were performed using a SEC column (Waters, ACQUITY UPLC Protein BEH SEC Column, 125 Å, 1.7 µm, 4.6 mm × 150 mm, 1–80 kDa) equipped with the respective guard column (Waters, ACQUITY UPLC Protein BEH SEC Guard Column, 125 Å, 1.7 µm, 4.6 mm × 30 mm, 1–80 kDa). The runtime was set to 15 min for a single run.

**Table 2:** Instrumental parameters for ICP–MS and SEC–ICP–MS measurements including chromatographic conditions

	ICP–MS Agilent 7500ce	SEC–ICP–MS Agilent 8800
RF power/W	1560	1350
Cones	Ni	Ni
Registered isotopes	^101^Ru, ^102^Ru, ^115^In, ^185^Re, ^189^Os, ^192^Os	^48^SO, ^72^Fe, ^101^Ru, ^102^Ru, ^189^Os, ^192^Os, ^206^OsO, ^224^OsO_2_, ^240^OsO_3_, ^256^OsO_4_
Dwell time/s	0.3	–
Replicates	10	1
Carrier gas (Ar)/dm^3^ min^−1^	0.92–0.97	1.1
Make up gas (Ar)/dm^3^ min^−1^	0.22–0.27	–
Plasma gas (Ar)/dm^3^ min^−1^	15	15
Reaction gas (O_2_)/cm^3^ min^−1^	–	0.3 (30%)
HPLC column	–	ACQUITY UPLC Protein BEH SEC, 125 Å, 1.7 µm, 4.6 mm × 150 mm
Eluent	–	50 mM CH_3_COONH_4_ (pH 6.8)
Flow rate/cm^3^ min^−1^	–	0.3
Injection volume/mm^3^	–	2
Column temperature/°C	–	30
Autosampler temperature/°C	–	4

### SEC calibration markers

Ferritin (2.8 µM, 440 kDa), albumin (1 µM, 66 kDa), ovalbumin (1 µM, 43 kDa), and l-methionine (1.6 µM, 149 Da) were used as mass calibration markers of the column.

### Incubation studies with human serum

Compounds **1** and **2** were dissolved in DMSO (approximately 4 mM). They were sequentially diluted with PBS (1:20), human serum (1:20), and mobile phase (1:20) to a final concentration of approx. 0.5 µM. Test samples were taken after 0, 30 min, 1, 2, 6, and 26 h incubation time at 37 °C in the dark.

### Assessment of cytotoxicity in the NCI-60 cancer cell line panel

The osmium compound **2** (NSC No. 776416) was sent to the National Institute of Health (USA) for evaluation in the NCI-60 cancer cell line panel. The compound was evaluated with respect to growth inhibition at a single concentration corresponding to 10 µM. A COMPARE 170 analysis was performed with the single-dose activity profile.

## References

[1] Szpunar J, Lobinski R, Prange A (2003). Appl Spectrosc.

[2] Montes-Bayón M, DeNicola K, Caruso JA (2003). J Chromatogr A.

[3] Gammelgaard B, Stürup S, Moller C (2012) Inductively coupled plasma–mass spectrometry for analysis of metal-containing pharmaceuticals. In: Lyubimov AV (ed) Encyclopedia of drug metabolism and interactions, vol 6. Wiley, pp 1–25

[4] Matz SG, Elder RC, Tepperman K (1989). J Anal At Spectrom.

[5] Groessl M, Terenghi M, Casini A, Elviri L, Lobinski R, Dyson PJ (2010). J Anal At Spectrom.

[6] Kratz F, Elsadek B (2012). J Control Release.

[7] Miele E, Spinelli GP, Miele E, Tomao F, Tomao S (2009). Int J Nanomed.

[8] Warnecke A, Fichtner I, Garmann D, Jaehde U, Kratz F (2004). Bioconjugate Chem.

[9] Pichler V, Mayr J, Heffeter P, Domotor O, Enyedy EA, Hermann G, Groza D, Kollensperger G, Galanksi M, Berger W, Keppler BK, Kowol CR (2013). Chem Commun.

[10] Mayr J, Heffeter P, Groza D, Galvez L, Koellensperger G, Roller A, Alte B, Haider M, Berger W, Kowol CR, Keppler BK (2017). Chem Sci.

[11] Hanif M, Nazarov AA, Legin A, Groessl M, Arion VB, Jakupec MA, Tsybin YO, Dyson PJ, Keppler BK, Hartinger CG (2012). Chem Commun.

[12] Mandal R, Kalke R, Li X-F (2004). Chem Res Toxicol.

[13] Bell DN, Liu JJ, Tingle MD, McKeage MJ (2006). J Chromatogr B.

[14] Mandal R, Sawyer MB, Li X-F (2006). Rapid Commun Mass Spectrom.

[15] Esteban-Fernandez D, Canas B, Pizarro I, Palacios MA, Gomez-Gomez MM (2007). J Anal At Spectrom.

[16] Xie R, Johnson W, Rodriguez L, Gounder M, Hall GS, Buckley B (2007). Anal Bioanal Chem.

[17] Esteban-Fernandez D, Verdaguer JM, Ramirez-Camacho R, Palacios MA, Gomez-Gomez MM (2008). J Anal Toxicol.

[18] Koellensperger G, Stefanka Z, Meelich K, Galanski M, Keppler BK, Stingeder G, Hann S (2008). J Anal At Spectrom.

[19] Falta T, Koellensperger G, Standler A, Buchberger W, Mader RM, Hann S (2009). J Anal At Spectrom.

[20] García Sar D, Montes-Bayón M, Blanco González E, Sierra LM, Aguado L, Comendador MA, Koellensperger G, Hann S, Sanz-Medel A (2009). Anal Chem.

[21] Bell DN, Liu JJ, Tingle MD, Rattel B, Meyer TU, McKeage MJ (2008). Clin Exp Pharmacol Physiol.

[22] Ip V, McKeage MJ, Thompson P, Damianovich D, Findlay M, Liu JJ (2008). J Anal At Spectrom.

[23] Hann S, Falta T, Boeck K, Sulyok M, Koellensperger G (2010). J Anal At Spectrom.

[24] Sulyok M, Hann S, Hartinger CG, Keppler BK, Stingeder G, Koellensperger G (2005). J Anal At Spectrom.

[25] Hermann G, Heffeter P, Falta T, Berger W, Hann S, Koellensperger G (2013). Metallomics.

[26] Theiner S, Grabarics M, Galvez L, Varbanov HP, Sommerfeld N, Galanski M, Keppler BK, Koellensperger G (2018). Dalton Trans.

[27] Meier-Menches SM, Gerner C, Berger W, Hartinger CG, Keppler BK (2018). Chem Soc Rev.

[28] Bytzek AK, Boeck K, Hermann G, Hann S, Keppler BK, Hartinger CG, Koellensperger G (2011). Metallomics.

[29] Loreti V, Bettmer J (2004). Anal Bioanal Chem.

[30] Miles DR, Mesfin M, Mody TD, Stiles M, Lee J, Fiene J, Denis B, Boswell GW (2006). Anal Bioanal Chem.

[31] Sarmiento-González A, Encinar JR, Cantarero-Roldán AM, Marchante-Gayón JM, Sanz-Medel A (2008). Anal Chem.

[32] Rodríguez-Fariñas N, Gomez-Gomez MM, Camara-Rica C (2008). Anal Bioanal Chem.

[33] Meier SM, Hanif M, Adhireksan Z, Pichler V, Novak M, Jirkovsky E, Jakupec MA, Arion VB, Davey CA, Keppler BK, Hartinger CG (2013). Chem Sci.

[34] Meier SM, Kreutz D, Winter L, Klose MHM, Cseh K, Weiss T, Bileck A, Alte B, Mader JC, Jana S, Chatterjee A, Bhattacharyya A, Hejl M, Jakupec MA, Heffeter P, Berger W, Hartinger CG, Keppler BK, Wiche G, Gerner C (2017). Angew Chem Int Ed.

[35] Klose MHM, Hejl M, Heffeter P, Jakupec MA, Meier-Menches SM, Berger W, Keppler BK (2017). Analyst.

[36] Klose MHM, Theiner S, Kornauth C, Meier-Menches SM, Heffeter P, Berger W, Koellensperger G, Keppler BK (2018). Metallomics.

[37] Hartinger CG, Groessl M, Meier SM, Casini A, Dyson PJ (2013). Chem Soc Rev.

[38] Bytzek AK, Koellensperger G, Keppler BK, Hartinger CG (2016). J Inorg Chem.

[39] Delord J-P, Umlil A, Guimbaud R, Grégoire N, Lafont T, Canal P, Bugat R, Chatelut E (2003). Cancer Chemother Pharmacol.

[40] Shoemaker RH (2006). Nat Rev Cancer.

[41] Larsen IK, Cornett C, Karlsson M, Sahlin M, Sjoberg BM (1992). J Biol Chem.

[42] Witte RS, Hsieh P, Elson P, Oken MM, Trump DL (1996). Investig New Drugs.

[43] Subtel’na I, Atamanyuk D, Szymańska E, Kieć-Kononowicz K, Zimenkovsky B, Vasylenko O, Gzella A, Lesyk R (2010). Bioorg Med Chem.

